# Expression, Characterization, and Cellular Localization of Knowpains, Papain-Like Cysteine Proteases of the *Plasmodium knowlesi* Malaria Parasite

**DOI:** 10.1371/journal.pone.0051619

**Published:** 2012-12-12

**Authors:** Rajesh Prasad, Atul, Awakash Soni, Sunil Kumar Puri, Puran Singh Sijwali

**Affiliations:** 1 CSIR-Centre for Cellular and Molecular Biology, Hyderabad, Andhra Pradesh, India; 2 CSIR-Parasitology Division, Central Drug Research Institute, Lucknow, Uttar Pradesh, India; Université Pierre et Marie Curie, France

## Abstract

Papain-like cysteine proteases of malaria parasites degrade haemoglobin in an acidic food vacuole to provide amino acids for intraerythrocytic parasites. These proteases are potential drug targets because their inhibitors block parasite development, and efforts are underway to develop chemotherapeutic inhibitors of these proteases as the treatments for malaria. *Plasmodium knowlesi* has recently been shown to be an important human pathogen in parts of Asia. We report expression and characterization of three *P. knowlesi* papain-like proteases, termed knowpains (KP2-4). Recombinant knowpains were produced using a bacterial expression system, and tested for various biochemical properties. Antibodies against recombinant knowpains were generated and used to determine their cellular localization in parasites. Inhibitory effects of the cysteine protease inhibitor E64 were assessed on *P. knowlesi* culture to validate drug target potential of knowpains. All three knowpains were present in the food vacuole, active in acidic pH, and capable of degrading haemoglobin at the food vacuolar pH (≈5.5), suggesting roles in haemoglobin degradation. The proteases showed absolute (KP2 and KP3) to moderate (KP4) preference for peptide substrates containing leucine at the P2 position; KP4 preferred arginine at the P2 position. While the three knowpains appear to have redundant roles in haemoglobin degradation, KP4 may also have a role in degradation of erythrocyte cytoskeleton during merozoite egress, as it displayed broad substrate specificity and was primarily localized at the parasite periphery. Importantly, E64 blocked erythrocytic development of *P. knowlesi*, with enlargement of food vacuoles, indicating inhibition of haemoglobin hydrolysis and supporting the potential for inhibition of knowpains as a strategy for the treatment of malaria. Functional expression and characterization of knowpains should enable simultaneous screening of available cysteine protease inhibitor libraries against knowpains for developing broadly effective compounds active against multiple human malaria parasites.

## Introduction

Malaria remains one of the most important infectious diseases in the world; it caused an estimated 216 million infections and 0.66 million deaths in 2010 [1]. *Plasmodium falciparum* and *Plasmodium vivax* are the two major human malaria parasites; *P. falciparum* is responsible for over 90% of malaria-related deaths. Several recent reports of *Plasmodium knowlesi* infections in humans further aggravate the malaria control situation. *P. knowlesi*, which was considered a malaria pathogen of only macaque monkeys until recently, has been shown to be responsible for several cases of severe and fatal malaria in Southeast Asia [2], [3], [4]. One of the biggest challenges to combating malaria is increasing resistance of the parasites to most available antimalarials. Thus, there is an urgent need for new antimalarial drugs [5], [6]. Papain-like cysteine proteases of malaria parasites are leading antimalarial drug targets because their inhibitors kill the parasite both in culture and in animal models [7], [8], [9], [10]. The best characterized cysteine proteases are those of the most virulent human malaria parasite, *P. falciparum*, known as falcipains [10], and several drug discovery efforts are underway to develop inhibitors of falcipains for the treatments of malaria [11], [12]. However, these drug discovery projects should ideally be extended to the homologous proteases of *P. vivax* and *P. knowlesi* to develop common inhibitors of these proteases as a broadly effective antimalarial therapy. Homologous *P. vivax* cysteine proteases, known as vivapains, have been characterized [13], [14], and homologs in *P. knowlesi* need to be characterized to augment ongoing falcipain-based drug development projects.

Multiple inhibitory effects of cysteine protease inhibitors on malaria parasites suggest multiple functions of parasite cysteine proteases, including a key role in haemoglobin degradation during erythrocytic development and processing of host and parasite proteins [10], [15], [16], [17], [18]. While developing inside the erythrocyte, malaria parasites take up and degrade haemoglobin in a lysosome-like organelle, known as the food vacuole, to obtain amino acids and maintain osmotic stability [19], [20], [21]. Proteases of cysteine, aspartic, metallo, and aminopeptidase classes appear to jointly participate in this process [7], [22], [23], [24], [25], [26]. A large body of literature indicates that falcipains are the major haemoglobin degrading enzymes in *P. falciparum* [10]. There are four falcipains: falcipain-1 (FP1), falcipain-2 (FP2), falcipain-2′ (FP2′), and falcipain-3 (FP3). FP1 shares 28–30% sequence identity with other three falcipains; FP2 and FP2′ are 92% identical and share 54% identity with FP3. All falcipains are expressed during the erythrocytic stage of parasite development, efficiently degrade haemoglobin at the food vacuolar pH (≈pH 5.5), and are inhibited by cysteine protease inhibitors that block haemoglobin degradation and parasite development [27], [28], [29], [30], [31], [32]. FP2 and FP3 are located in the food vacuole, consistent with roles of falcipains in haemoglobin degradation. Disruption of the FP2 gene caused accumulation of undegraded haemoglobin and enlargement of the food vacuole, a characteristic of cysteine protease inhibitor-treated parasites, confirming that FP2 is a major haemogobin degrading enzyme [22], [26]. Compared to wild type parasites, the FP2 knockout parasites were almost twice as susceptible to cysteine protease inhibitors and over a thousand fold more susceptible to the aspartic protease inhibitor pepstatin, supporting joint and redundant roles of different classes of proteases in haemoglobin degradation.

Cysteine protease inhibitors block egress of merozoites by inhibiting rupture of mature schizont-infected erythrocytes, suggesting a role of falcipains in this process [33], [34]. Recombinant FP2 has also been shown to cleave erythrocyte membrane proteins, suggesting that it mediates merozoite egress [35], [36]. Knockout of FP1 and FP2′ genes did not affect erythrocytic stage development [22], [37]. Conversely, a role for FP1 in invasion of erythrocytes by merozoites has been suggested based on inhibition of this process by a FP1-specific inhibitor [38]. In other studies, FP1 knockout parasites produced 70–90% fewer oocysts than wild type parasites, indicating an important role for FP1 in mosquito stages [39]. Thus, as individual knockouts of FP1, FP2, and FP2′ genes resulted in viable parasites, these three proteases may have redundant functions. On the other hand, the FP3 gene could not be knocked out but was easily replaced by a functional allele, suggesting that FP3 is essential for parasite development [22]. Taken together, as FP2 has a key role in haemoglobin hydrolysis and FP3 is essential, FP2 and FP3 are the *P. falciparum* papain-like cysteine proteases of greatest interest as targets for drug discovery.

A number of drug discovery programs are underway to develop potent peptide, peptidomimetic, and nonpeptide inhibitors of FP2 and FP3 [11], [12]. The availability of crystal structures [40], [41], [42], a variety of small molecule chemotypes [11], [12], and extensive biochemical studies of both FP2 and FP3 strongly aid the ongoing inhibitor development programs. Notably, unlike the major host homologs cathepsin L and B that prefer phenylalanine to leucine at the P2 position in substrates and inhibitors, falcipains and their characterized Plasmodium homologs prefer leucine at this position [13], [14], [27], [28], [43], [44], [45], [46]. This selectivity for a P2 leucine residue may be exploited for optimizing inhibitors of falcipains and related parasite proteases.

A single clearly identifiable FP1 homolog is present in all malaria parasites, whereas human and monkey malaria parasites have three and mouse malaria parasites have only one homolog of the remaining three falcipains (FP2, FP2′, and FP3), which will be referred to as FP2/3 homologs henceforth. The FP2/3 homologs of the human malaria parasite *P. vivax*, known as vivapains (VX2-4), and the mouse malaria parasites *Plamodium berghei* (called bergheipain-2 or BP2) and *Plasmodium vinckei* (called vinckeipain-2 or VP2) have been characterized [13], [14], [46]. All three vivapains (VX2-4), BP2, and VP2 are biochemically similar to FP2 and FP3, as they are also optimally active at the food vacuolar pH, degrade haemoglobin and selected erythrocyte cytoskeleton proteins, and prefer peptide substrates/inhibitors containing leucine at the P2 position [13], [14], [46], [47]. However, we have only a limited understanding of the biology of these proteases; VP2 and VX4 are expressed in erythrocytic stage parasites, and VX4 is present both in the food vacuole and in the cytoplasm of *P. vivax* trophozoites. Lack of an in vitro culture system of *P. vivax* is a major obstacle to study vivapain biology and assess effectiveness of their inhibitors against the parasite. *P. vivax* is evolutionarily closest to monkey malaria parasites, including *P. knowlesi* [48], which can be propagated in vitro and also offers efficient functional genomics studies [49]. Hence, *P. knowlesi* may serve as a convenient model system to study *P. vivax*.

Frequent mixed Plasmodium species infections necessitate treatments with drugs that are broadly effective against all human malaria parasites [50]. For falcipain inhibitors to be effective as a broad antimalarial therapy, ongoing drug discovery efforts need to be extended to vivapains and the FP2/3 homologs of *P. knowlesi*. As the papain-like cysteine proteases of *P. knowlesi* were not previously characterized, the present study describes functional expression, biochemical properties, and cellular localization of three *P. knowlesi* proteases, which we have termed knowpain-2 (KP2), knowpain-3 (KP3), and knowpain-4 (KP4).

## Materials and Methods

### Materials

All biochemicals were from Sigma, Calbiochem, and Serva. Plasmid isolation kits were from Qiagen or MACHEREY*-*NAGEL. Cell culture reagents were from Lonza and Invitrogen. Restriction endonucleases and DNA modifying enzymes were from New England Biolabs. Secondary antibodies and 4′,6-diamidino-2-phenylindole (DAPI) were from Invitrogen. Peptide substrates were from Bachem, and protease inhibitors were from Serva. *P. knowlesi* genomic DNA was obtained from the Malaria Research and Reference Reagent Resource Centre (MR4).

### Sequence Analysis of Knowpains

FP2 (accession number XP_001347836) and FP3 (XP_001347833) sequences were used as queries in BLAST searches of the PlasmoDB database (http://plasmodb.org) to identify their homologs in *P. knowlesi*. Amino acid sequences of knowpains were used as queries in BLAST searches against the MEROPS peptidase database (http://merops.sanger.ac.uk/), the conserved domain database (CDD) at NCBI (http://www.ncbi.nlm.nih.gov/Structure/cdd/wrpsb.cgi), and Pfam database (http://pfam.sanger.ac.uk/) to identify conserved domains and catalytic residues. Sequence alignment of knowpains with vivapains (VX2, VX3, and VX4), bergheipain (BP2), and falcipains (FP2 and FP3) was performed by MULTALIN (http://pbil.ibcp.fr/htm/index.php). Transmembrane regions were predicted using the PHDhtm program (http://npsa-pbil.ibcp.fr). Functional motifs were predicted based on conservation of the characterized motifs. For phylogenetic tree construction, mature protease domains, beginning with the first N-terminus conserved DWR motif of papain-like cysteine proteases of parasitic protozoa and human cathepsins (K, L, and S), were first aligned using the ClustalW program and then subjected to phylogenetic tree analysis using the Neighbor Joining algorithm.

### Expression and Purification of Knowpains

Portions of the knowpain genes corresponding to the C-terminus of prodomains and the entire mature protease domains (KP2, 336 amino acid residues; KP3, 362 amino acid residues; and KP4, 311 amino acid residues) were amplified from *P. knowlesi* genomic DNA using Phusion DNA polymerase and primers (KP2: PK60expF 5′- TTT**GGATCC**GCCGACTCCAGATTTTTGATGA CGAAT-3′ and PK60expR 5′-TTC**CTGCAG**TTTCCCCTTCTTCCTCACAAAAAC-3′; KP3: PK50expF 5′-TGC**GGATCC**AGTGTTGGTCCTTACAGGATGAAT-3′ and PK50expR 5′-GTA**CTGCAG**C GTGAGGGTTAAATTTCTTCAATTAA-3′; KP4: PK40expF 5′-CAT**GGATCC**GGAAAAAATATCAAACTCCAGATGAA-3′ and PK40expR 5′-CAC**CTGCAG**CTAGTCAAGCAGGGGGACATA-3′) containing BamH1 (forward primers) and Pst1 (reverse primers) sites. The PCR products were purified and 3′-A overhangs were added to the PCR products using Taq DNA polymerase and deoxynucleotides at 70°C for 30 min. The tailed-products were cloned using the TOPO® TA Cloning® kit (Invitrogen) to obtain knowpain constructs (pTOPO-KP2, pTOPO-KP3, and pTOPO-KP4), and the inserts were sequenced to confirm desired sequences. The inserts were excised from the confirmed knowpain clones with BamHI-PstI and cloned into similarly digested pRSETA (KP2 and KP4) and pET-32a (KP3) plasmids to obtain expression constructs (pRSETA-KP2, pRSETA-KP4, and pET32a-KP3), which were then used to transform BL21(DE3) *Escherichia coli* cells. pRSETA provides a His-tag and pET-32a provides a thioredoxin/His-tag at the N-terminus of the expressed proteins, which allow purification of recombinant proteins by nickel- nitrilotriacetic acid (Ni-NTA) chromatography.

The knowpain expression clones were grown to mid-log phase and induced with isopropyl-1-thio-β-D-galactopyranoside (IPTG, 1 mM final) for 4 h at 37°C with shaking at 200 rpm, and the cultures were centrifuged at 6000 g for 5 min at 4°C. The induced pellets were suspended in native lysis buffer (50 mM NaH_2_PO_4_, 100 mM NaCl, pH 8.0, lysozyme at 1 mg/ml; 5 ml buffer/g pellet) and incubated for 30 min at 4°C, and then sonicated at 4°C for 4–5 min using pulses of 9 seconds on/off at 20% amplitude (Sonics). The lysates were centrifuged at 27000 g for 15 min at 4°C, and supernatants containing soluble proteins and pellets with insoluble proteins/inclusion bodies were separated. The pellets were suspended in binding buffers (KP2∶8M Urea, 50 mM NaH_2_P0_4_, 250 mM NaCl, 10 mM imidazole, 1% Triton X-100 and 5 mM β-ME, pH 8.0; KP3∶8M Urea, 50 mM NaH_2_P0_4_, 250 mM NaCl, 10 mM imidazole, 1% Triton X-100 and 1 mM β-ME, pH 8.0; KP4∶8M Urea, 50 mM NaH_2_P0_4_, 250 mM NaCl, 10 mM imidazole, pH 8.0; 5 ml buffer/1.0 g pellet) and incubated at room temperature for 30 min with gentle shaking. The lysates were centrifuged at 27000 g for 30 min, pellets were discarded and supernatants were incubated with Ni-NTA resin (preequilibriated with corresponding urea buffers; 0.5 ml resin slurry/1.0 g weight of the initial pellet) for 30 min at room temperature. The suspensions were transferred to columns and allowed to run under gravity. The resins for KP2 and KP4 were first washed with 25× column volume of the respective binding buffer, then washed with urea buffer (8M Urea, 50 mM NaH_2_P0_4_, 250 mM NaCl, pH 8.0) containing 25–50 mM imidazole to remove nonspecific proteins, and proteins were eluted with 250 mM imidazole in the urea buffer. Elution fractions were run in 12% SDS-PAGE, and the gel was stained with coomassie blue to check for the presence and purity of recombinant knowpains. For KP3, the resin was washed with urea buffer (8M Urea, 50 mM NaH_2_P0_4_, pH 8.0), bound proteins was eluted with 250 mM imidazole in the same urea buffer, elution fractions were run in 12% SDS-PAGE, and the gel was stained with coomassie blue to check for the presence and purity of KP3. As the purified KP3 also contained low levels of additional proteins, it was further purified by Q-sepharose chromatography under denaturing conditions. Briefly, β-ME (1 mM final) and Triton X-100 (1% v/v final) were added to the protein sample, which was then incubated with Q-sepharose (preequilibriated with 8M Urea, 50 mM NaH_2_P0_4_, 1% Triton X-100, 1 mM β-ME, pH 8.0). The resin was washed twice with 25× column volume of urea buffer (8M Urea, 50 mM NaH_2_P0_4,_ 10 mM NaCl, pH 8.0), and proteins were eluted with 200 mM NaCl (in 8M Urea, 50 mM NaH_2_P0_4,_ pH 8.0). Elution fractions were run in 12% SDS-PAGE, the gel was stained with coomassie blue to check for the presence and purity of KP3, and elution fractions containing pure KP3 were pooled and refolded.

### Refolding of Knowpains

As the above purified knowpains were denatured and inactive, they needed to be refolded into active forms. For optimization of refolding, each knowpain sample was adjusted to 0.25 mg/ml and dialyzed using a 10 kDa cut-off dialysis membrane for 16 hr at 4°C against 100 fold excess of six different buffers (A: 100 mM Tris, 1 mM EDTA, 1 mM GSH, 0.5 mM GSSG, pH 8.0; B: A with 20% glycerol; C: A with 250 mM L-arginine; D: A with 20% sucrose; E: A with 20% sucrose and 250 mM L-arginine; F: A with 20% glycerol and 250 mM L-arginine). The proteins were further dialysed against 10 mM Tris pH 7.5 at 4°C with one change of buffer after 4 hours. To compare refolding efficiencies in different buffers, 50 µl of each dialyzed sample was added to an assay buffer (100 mM sodium acetate, 10 mM dithiothreitol (DTT), pH 5.5, and 25 µM Z-LR-AMC) and substrate hydrolysis was monitored at room temperature for 30 min as described below. For each knowpain, the dialysis buffer of the sample that showed maximum activity was chosen as the optimum refolding buffer (buffer A for KP2, buffer C for KP3, and buffer D for KP4). Refolding was further optimized by dialysing denatured knowpains against the optimized buffers at different ratios (25, 50, and 100 fold excess of buffer) for 16 hours at 4°C, and then against 10 mM Tris pH 7.5 at 4°C with one change of buffer after 4 hours. Refolding efficiencies of the three conditions were assessed by measuring Z-LR-AMC hydrolysis with equal amounts of dialyzed samples as described below. The optimum refolding buffers were used at 50 fold (for KP2) and 25 fold (KP3 and KP4) excess.The optimized refolding methods were used for large-scale refolding, and refolded samples were concentrated to 1–2 ml using Ultracel-10k (Millipore) at 4°C.

For processing into mature forms, the concentrated refolded knowpain samples were acidified, reduced (KP2 and KP4∶100 mM sodium acetate, pH 5.5–6.0, 10 mM DTT; KP3∶25 mM sodium acetate, pH 5.5–6.0, 2 mM GSH), and centrifuged at 27,000 g for 15 min. The supernatants were transferred to fresh tubes and pellets were discarded. The supernatant of the KP2 sample was dialyzed against 500 fold excess of processing buffer (100 mM sodium acetate, 10 mM DTT, pH 6.0) for 3 hours at room temperature, and then passed through a Q-Sepharose column (equilibrated with 100 mM sodium acetate, pH 6.0, 5 mM NaCl; Sigma) at 4°C. The column was washed with 10-bed volumes of the same buffer, and the protein was eluted with 250 mM NaCl (in 100 mM sodium acetate, pH 6.0). The elution fractions were assessed for hydrolysis of Z-LR-AMC as described below; fractions with activity were pooled and concentrated to 1.0 ml using a 30 kDa cut off Centricon (Millipore), an equal volume of glycerol was added to the sample, and the enzyme was stored at −20°C. The supernatant of the KP3 sample was incubated at 37°C for 4 hours, aliquots were taken out at 1 hour intervals and run on 12% SDS-PAGE to monitor processing, KP3 was purified from the remaining sample using Q-Sepharose as described for KP2, and the enzyme was stored with 50% glycerol at −20°C. The supernatant of KP4 was dialyzed against 500 fold excess of processing buffer (100 mM sodium acetate, 10 mM DTT, pH 6.0) for 3 hours at room temperature, an equal volume of glycerol was added to the processed sample and the enzyme was stored at −20°C. The processed knowpain samples together with total lysates of uninduced and induced cells, soluble and urea buffer-solubilized insoluble fractions of induced cells, and purified Ni-NTA and/or ion exchange samples were resolved by 12% SDS-PAGE and then stained with coomassie blue to assess integrity and purity of recombinant knowpains.

### Characterization of Recombinant Knowpains

Routine activity assays of knowpains were carried out in 200 µl sodium acetate buffer (100 mM sodium acetate, pH 5.5, 10 mM DTT) containing the enzyme (250 nM KP2, 100 nM KP3, and 50 nM KP4) and the fluorogenic peptide substrate Z-LR-AMC (25 µM final). Enzyme activity was measured by monitoring the release of AMC upon substrate hydrolysis over 15–30 min at 37°C using an Infinite M200 (TECAN) or SpectraMax M5 (Molecular Devices) microplate reader (excitation 355 nm; emission 460 nm), and expressed as relative fluorescence units/minute.

Concentration of active enzyme in knowpain samples was determined by active-site titration with *trans*-epoxysuccinyl-L-leucylamido-(4-guanidino) butane (E-64; for KP3 and KP4) or leupeptin (for KP2). Briefly, enzymes were incubated with DMSO (1% final) or varying concentrations of inhibitors in sodium acetate buffer for 30 min at room temperature, Z-LR-AMC was added (25 µM final) to the reaction, and the remaining enzyme activity was monitored for 30 min at 37°C as described for routine activity assays. Enzyme activities for DMSO and inhibitor-containing reactions were plotted against inhibitor concentrations to determine the enzyme concentration.

For pH-dependent activity, assays of knowpains were carried out in different pH buffers (100 mM sodium acetate, pH 4.5, 5.0, 5.5; 25 mM Bis-Tris, pH 6.5; and 25 mM Tris, pH 7.0, 7.5 and 8.0) containing Z-LR-AMC (25 µM final) and enzymes as described above. Activities at different pHs were normalized against the maximum activity, and plotted against pH.

The effect of reducing agents on enzyme activity was determined as described for routine activity assays except that the assay buffer contained different concentrations of DTT or GSH. Activities at different concentrations of DTT or GSH were normalized against the maximum activity, and plotted against the concentrations of reducing agents.

For inhibition assays, knowpains (75 nM KP2, 100 nM KP3, and 10 nM KP4) were incubated with DMSO as a control (0.75%) or indicated inhibitors (E64, leupeptin, and pepstatin, each at 10 µM; 1 mM PMSF; and 5 mM EDTA) in 200 µl sodium acetate buffer for 5 min at room temperature. Z-LR-AMC was added and activity was measured as described for routine activity assays. Activities were expressed as percent of the control and plotted against corresponding inhibitors.

For pH stability, knowpains (75 nM KP2, 100 nM KP3, and 10 nM KP4) were incubated in a pH 5.5 (25 mM sodium acetate) or pH 7.5 (25 mM Tris) buffer containing 1 mM GSH and 1 mM EDTA at 37°C for 3 hours. 10 µl samples were collected at 30 min intervals and added to 190 µl of the assay buffer (100 mM sodium acetate, pH 5.5, 10 mM DTT, 25 µM Z-LR-AMC), and enzyme activities were monitored for 15 min at 37°C as described for routine assays. For each knowpain, enzyme activities at different time intervals were normalized as percentage of the initial activity.

### Enzyme Kinetics

Rates of hydrolysis of fluorogenic peptide substrates (Z-LR-AMC, Z-FR-AMC, and Z-RR-AMC) by knowpains were determined by incubating increasing concentrations of substrates with individual enzymes (250 nM KP2, 100 nM KP3, and 50 nM KP4) in 200 µl assay buffer (100 mM sodium acetate pH 5.5 and 10 mM DTT) and monitoring hydrolysis of the substrate for 30 min at 37°C as described for routine activity assays. For KP2, rates of hydrolysis of substrates were also determined at pH 7.5 (50 mM Tris and 10 mM DTT). The relative fluorescence units/minute were converted into the rate of product formation (concentration of AMC released) using a standard curve of the fluorescence of free AMC versus its concentration. The rate of product formation was used to determine *K*_m_ and *V*_max_ using the ORIGIN program as has been described [28].

### Haemoglobin Hydrolysis

Knowpains (50 nM) were incubated with haemoglobin (80 µg) in 480 µl 100 mM sodium acetate buffer (pH 5.5 with 5 mM DTT or 2 mM GSH) at 37°C for 3 hour. 60 µl aliquots were taken at various time intervals, the reaction was stopped by the addition of 20 µl of 4× SDS-PAGE reducing sample buffer, 32 µl of each sample was run in 16% SDS-PAGE and the gel was stained with coomassie blue. KP2 was also assessed for haemoglobin hydrolysis at neutral pH (25 mM Tris, pH 7.0).

### Degradation of Erythrocyte Cytoskeleton Proteins

Human blood was collected aseptically in a heparinized tube and centrifuged at 350 g for 10 min. The plasma along with the buffy coat was aspirated off, and the RBC pellet was suspended in PBS (10× of the packed cell volume), which was then centrifuged at 350 g for 10 minutes. The supernatant along with the buffy coat was aspirated off, and the pellet was washed again twice with PBS. The washed RBC pellet (1 ml packed cell volume) was suspended in 100 ml of a hypotonic buffer (5 mM MgCl_2_ in 5 mM phosphate buffer, pH 8), incubated at 4°C for 10 min, and the lysate was centrifuged at 18000 g for 10 minutes at 4°C. The supernatant was discarded, and the erythrocyte ghost pellet was washed several times with the hypotonic buffer until the pellet became white. The erythrocyte ghost pellet was suspended in 2.5 ml of 25 mM Tris-Cl, pH 7.5, and this membrane preparation was stored at −20°C or used for hydrolysis of membrane proteins by knowpains. Protein concentration of the membrane preparation was determined by using the Bio-Rad protein assay reagent (Bio-Rad). 30 µg of the membrane preparation was incubated with knowpains (200 nM) or without any enzyme (control) in 150 µl of 50 mM Tris-Cl, pH 7.5 for 1 hour at 37°C, and then reaction was stopped by adding 50 µl of 4×SDS-PAGE reducing sample buffer. The samples were boiled for 5 min, centrifuged at 27216 g for 15 min at room temperature, and the supernatants were separated. Aliquots corresponding to 1.5 µg of the membrane preparation of each supernatant were resolved on 10% SDS PAGE, and then transferred onto a nitrocellulose membrane. The membrane was blocked with 5% nonfat dry milk in TBST (100 mM Tris-Cl, 150 M NaCl, 0.01% Tween-20, pH 7.5) for overnight, and incubated with antibodies against β actin (mouse antibodies at 1/800 dilution in blocking buffer; Santa Cruz Biotechnology, Cat No. sc-47778), spectrin α (mouse antibodies at 1/20000 dilution; Santa Cruz Biotechnology, Cat No. sc-271130), and spectrin β (mouse antibodies at 1/8000 dilution; Santa Cruz Biotechnology, Cat No. sc-374309). The membrane was washed with TBST, and then incubated with HRP-conjugated goat anti-mouse IgG (Invitrogen, Cat No. 62-6420; at 1/10000 dilution in blocking buffer), and the signal was developed using the SuperSignal West Pico Chemiluminescent kit (Pierce) on X-ray film.

### Generation of Antibodies

Processed (KP2 and KP4) and unprocessed (thioredoxin tagged-KP3) enzymes were inhibited with leupeptin (KP2) or E64 (KP3 and KP4) and used to immunize (100 µg/immunization) Wistar rats intraperitoneally with complete (day 0) or incomplete Freund’s adjuvant (days 15, 30, 60, 90, and 120). Blood was collected on days 105 and 135; serum was collected off the clot and evaluated for antibody titers against recombinant knowpains by western blotting. Final serum was obtained from the blood collected on day 140, and stored at −20°C with sodium azide (0.1% final). The KP3 antiserum was purified to remove antibodies against the thioredoxin-tag by ammonium sulfate fractionation and then incubation with recombinant thioredoxin. The anti-sera and purified IgG samples were stored at −20°C with sodium azide (0.1% final). The anti-sera and purified antibodies were checked for cross reactivity with recombinant knowpains by western and dot blotting as described in Figure S2 [28].

### Immunofluorescence Assay

Animals were housed in a CPCSEA (Committee for the Purpose of Control and Supervision of Experiment on Animals) registered animal facility as per the guidelines of the Indian National Science Academy, India for animal care and use in scientific research. Animals were maintained in cabin type isolators at standard environmental conditions (22–25°C, 40–70% humidity, and 12∶12 hour dark/light photoperiod). All animal experiments were carried out according to the protocol (IAEC 12/2012) approved by the Institutional Animal Ethics Committees (IAEC) of CDRI and CCMB. *P. knowlesi* was maintained in a rhesus monkey, and infection was monitored daily by examining Giemsa-stained blood smears of the monkey. Blood was collected at ring (for inhibition experiments) or late trophozoite/schizont (for immunofluorescence assays) stages in Alsever’s solution. For immunofluorescence, cells were washed with PBS, immobilized on poly L-lysine coated slides for 30 min at room temperature, and fixed (3% paraformaldehyde and 0.01% glutaraldehyde in PBS) for 60 min at room temperature. The fixed cells were permealized with 0.1% Triton-X 100 (in PBS), blocked (3% BSA in PBS), and incubated with primary antibodies (diluted in blocking buffer at 1/1000 for anti-KP2, 1/10 for anti-KP3, and 1/400 for anti-KP4). Unbound antibodies were washed off with PBS, and the cells were incubated with FITC-conjugated anti-rat IgG (diluted 1/1000 in blocking buffer) and DAPI (10 µg/ml PBS). Slides were washed with PBS and air dried in the dark. ProLong® Gold antifade reagent (Invitrogen) was added to the samples, and the samples were covered with a coverslip. The corners of the coverslip were sealed with nail polish. The cells were viewed under a 100× objective lens using a TCS SP5 Leica Confocal Microscope, and images were processed using LAS AF software.

### Effect of E64 on the Erythrocytic Stage Development

Blood was collected from a *P. knowlesi*-infected rhesus monkey, which had 0.5% infection with primarily ring stage parasites, in a tube containing Alsever’s solution. The blood was centrifuged, and the pellet containing infected erythrocytes was washed twice with RPMI 1640 medium. The washed pellet was suspended in complete medium (RPMI 1640 with 41.1 mg/litre hypoxanthine, 300 mg/litre glutamine, and 0.5% Albumax II) at 4% haematocrit. 100 µl of this suspension was added to wells of a 96 well plate containing 100 µl of complete medium with DMSO (0. 5%) or varying concentrations of E64. Two such plates were prepared, and incubated in a candle jar at 37°C for 20 or 30 hours (*P. knowlesi* takes ≈24 hour to complete its erythrocytic development). Thin smears of cultures from both the plates were stained with Giemsa and observed under a 100× objective lens using a bright field microscope. The control (DMSO) and E64-treated parasites were compared for the effect of E64 on parasite morphology, maturation, and multiplication. For studying morphology, images were taken under a 100× objective lens using a Zeiss AxioImager Z1 microscope fitted to an AxioCam CCD camera, and processed using Axiovision software. For maturation, the number of infected cells with 3 or more nuclei/300 infected cells was determined after 20 hours for each replicate, and the average of triplicate assessments was expressed as percentage of the control to determine the IC_50_ for E64. For effect on multiplication, the total number of parasite-infected cells/1000 cells was determined after 30 hours for each replicate, and the average of triplicate reads was expressed as percentage of the control to determine the IC_50_ for E64.

## Results

### Knowpains Belong to the FP2/3 Subfamily

**Figure 1:**
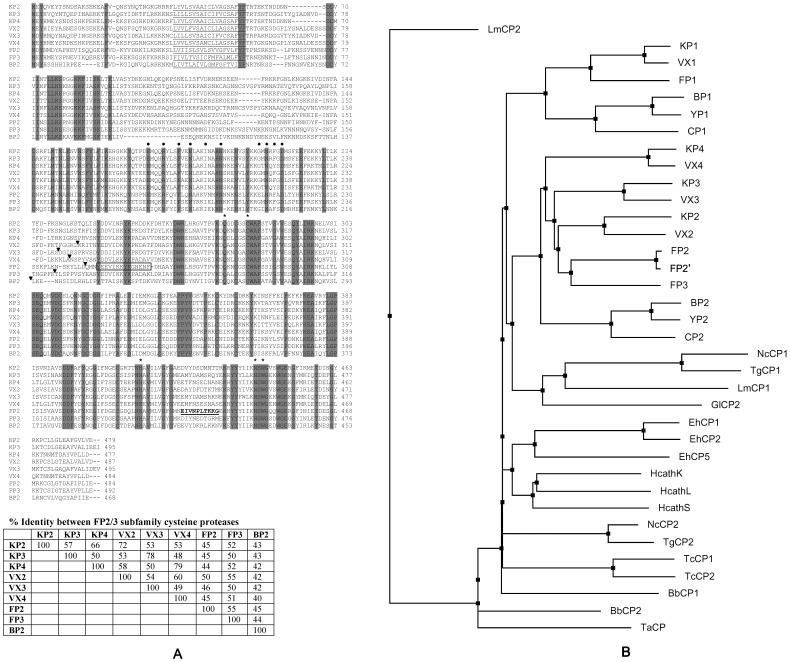
Sequence alignment of knowpains. A. The deduced amino acid sequences of knowpain-2 (KP2), knowpain-3 (KP3), knowpain-4 (KP4), vivapain-2 (VX2), vivapain-3 (VX3), vivapain-4 (VX4), falcipain-2 (FP2), falcipain-3 (FP3) and bergheipain (BP2) were aligned by using the multalin program. Predicted transmembrane domains are underlined, and ERFNIN and GNFD inhibitory motifs in the prodomain are labeled with filled circles. The positions of mature domain processing sites are indicated by arrowheads, and the catalytic amino acids are indicated by asterisks. The refolding domain and haemoglobin-binding motif of FP2 are boxed and underlined, respectively. The table shows % sequence identities within the FP2/3 subfamily based on the entire prodomain-mature protease regions. **B.** The phylogram was generated using the Neighbor Joining algorithm from the sequence alignment of mature protease domains (beginning with the conserved DWR motif to the end) of papain-like proteases of parasitic Protozoa and their selected human homologs (cathepsin L, cathepsin K, and cathepsin S). The accession numbers for various proteases are: *P. knowlesi* proteases knowpain-1 (KP1, XP_002260291), knowpain-2 (KP2, XP_002259153), knowpain-3 (KP3, XP_002259152), knowpain-4 (KP4, XP_002259151); *P. falciparum* proteases falcipain-1 (FP1, XP_001348727, falcipain-2 (FP2A XP_001347836), falcipain-2′ (FP2′, XP_001347832), falcipain-3 (FP3, XP_001347833); *P. vivax* proteases vivapain-1 (VX1, XP_001615807), vivapain-2 (VX2, XP_001615274), vivapain-3 (VX3, XP_001615273), vivapain-4 (VX4, XP_001615272); *P. berghei* proteases bergheipain-1 (BP1 XP_677643) and bergheipain-2 (BP2, PBANKA_093240); *P. chabaudi chabaudi* proteases chaubipain-1 (CP1, AAP43629) and chaubipain-2 (CP2, AAP43630); *P. yoelii yoelii* proteases yoelipain-1 (YP1, XP_729023) and yoelipain-2 (YP2, XP_726900); *Babesia bovis* proteases bovipain-1 (BV1, XP_001612131) and bovipain-2 (BV2, XP_001610695); *Entamoeba histolytica* cysteine protease-1 (EhCP1, Q01957), cysteine protease-2 (EhCP2, Q01958), cysteine protease-5 (EhCP5, CAA62835); *Giardia lamblia* cysteine protease-2 (GlCP2, EAA41050); *Leishmania major* cysteine protease-1 (LmCP1, AAB48119), cysteine protease-2 (LmCP2, AAB48120); *Neospora caninum* cysteine protease-1 (NcCP1, CCA30060) cysteine protease-2 (NcCP2, CBZ49954); *Theileria annulata* cysteine protease (TaCP, AAA30135); *Toxoplasma gondii* cysteine protease-1 (TgCP1, AAL60053), cysteine protease-2 (TgCP2, ABY58967); *Trypanosoma cruzi* cysteine protease-1 (TcCP1, AAA30181), cysteine protease-2 (TcCP2, AAC37213); human cathepsin L (HcathL), AAC23598), cathepsin S (HcathS, AAC37592) and cathepsin K (HcathK, P43235).

A search of the *P. knowlesi* genome sequence on PlasmoDB using FP2 and FP3 sequences as queries identified four closely related sequences, which were analyzed for the presence of conserved domains and compared with other papain-like cysteine proteases. The identified proteases were termed knowpains and designated as knowpain-1 (KP1), knowpain-2 (KP2), knowpain-3 (KP3), and knowpain-4 (KP4). KP1 showed 49% identity with FP1 and 26–28% identity with other three falcipains (FP2, FP2′, and FP3), indicating that it belongs to the FP1 subfamily. As FP1 is dispensable for erythrocytic stage parasite development and its functional expression has had limited success, KP1 was excluded from this study. KP2, KP3, and KP4 shared a typical prodomain-mature protease domain zymogen-like organization as seen in other papain-like proteases, with conservation of catalytic amino acid residues (Figure 1A). Additionally, unlike most papain-like proteases, all three knowpains contain unique features of the FP2/3 subfamily, which include an unusually large prodomain with an N-terminal hydrophobic region, a refolding domain in the beginning of the mature protease domain, and a haemoglobin-binding insert in the C-terminus between highly conserved catalytic histidine and aspargine residues. The putative mature protease domain of each knowpain showed maximum identity with one of the vivapains (72% identity between KP2 and VX2, 78% between KP3 and VX3, and 78% identity between KP4 and VX4), which allowed their designation according to the most similar vivapain. KP2, KP3, and KP4 showed slightly higher identities with FP3 (50–52%) than with FP2 (44–45%) and BP2 (41–42%). Compared to the mature protease regions, sequence identities of knowpains with other FP2/3 subfamily proteases across the entire prodomain-mature protease regions are slightly lower (Figure 1A). The prodomains of knowpains also contain ERFNIN and GNFD inhibitory motifs, which are present in the majority of cathepsin L-like proteases [51], [52], including proteases of the FP2/3 subfamily (Figure 1A) [53].

A phylogram was constructed based on the sequences of mature protease domains of papain-like cysteine proteases of parasitic protozoa and human cathepsins (K, L, and S) (Figure 1B). All Plasmodium proteases were clustered into two divergent branches: one containing all F2/3-like proteases, and another one with all FP1-like proteases. The clustering of Plasmodium proteases into two groups indicates two distinct subfamilies: a FP2/3 subfamily and a FP1 subfamily. The presence of two subfamilies is in agreement with higher identities between members of the same subfamily, eg the FP2/3 members share >42% sequence identity within the group and around 29% identity with members of the FP1 group, whereas the identity between FP1 group members is >45%. KP2, KP3, and KP4 were part of the FP2/3 group, and shared node with the most similar vivapain, which is in agreement with maximum similarity between knowpains and vivapains. Furthermore, within each subfamily, proteases of rodent malaria parasites were part of the same clade whereas the human and monkey malaria parasite proteases formed another clade. Homologous proteases from non Plasmodium protozoan parasites and human cathepsins were present on different branches.

### Production of Recombinant Knowpains

**Figure 2:**
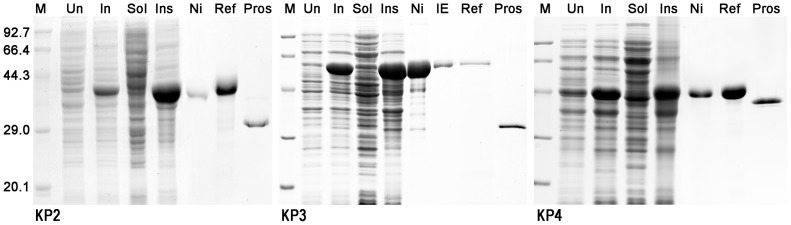
Production of recombinant knowpains. Knowpains were expressed in BL21(DE3) *E. coli* cells, purified from induced cells under denaturing conditions, refolded, and processed into mature proteases. For indicated knowpains (knowpain-2, KP2; knowpain-3, KP3; and knowpain-4, KP4) samples of the total lysates of uninduced (Un) and induced (In) cells, soluble (Sol) and insoluble (Ins) fractions of induced cells, Ni-NTA purified (Ni) and ion-exchange purified (IE) proteins, and refolded (Ref) and processed mature proteases (Pros) were resolved by 12% SDS-PAGE under reducing conditions. The gel was stained with coomassie blue. The same marker (M) was used in all cases, and molecular masses of markers are indicated in kDa; and the position of 29 kDa marker for the processed KP3 lane is indicated.

DNA fragments coding for the C-terminus regions of prodomains and complete mature domains of knowpains were expressed in BL21(DE3) *E. coli* cells with an N-terminus His-tag (KP2 and KP4) or thioredoxin/His-tag (KP3). Expression and cellular fractionation analyses of knowpain expression clones showed the presence of the expected sizes of proteins in IPTG-induced cell lysates, which were predominantly present as insoluble inclusion bodies (Figure 2). Knowpains were purified from inclusion bodies under denaturing conditions by Ni-NTA chromatography. Recombinant KP3 was further purified by ion exchange chromatography under denaturing conditions using Q-sepharose. The purified proteins were dialyzed against several buffers to optimize refolding conditions, which identified buffer A (100 mM Tris, pH 8.0, 1 mM EDTA, 1 mM GSH and 0.5 mM GSSG) for KP2, buffer C for KP3 (A with 250 mM L-arginine), and buffer D (A with 20% sucrose) for KP4 as optimum refolding buffers. Refolded KP2 and KP4 underwent processing to smaller sizes, presumably by virtue of their autocatalytic activity upon exposure to an acidic environment with DTT (Figure 2). KP3 did not undergo processing under the same conditions; rather, it rapidly lost most of its activity. Hence, KP3 was subjected to processing in an acidic environment with GSH (Figure S1). Each processed sample contained a predominant single protein, which was smaller than the parent recombinant protein, likely representing the mature protease after processing of the prodomain.

### Biochemical Properties of Knowpains

**Figure 3:**
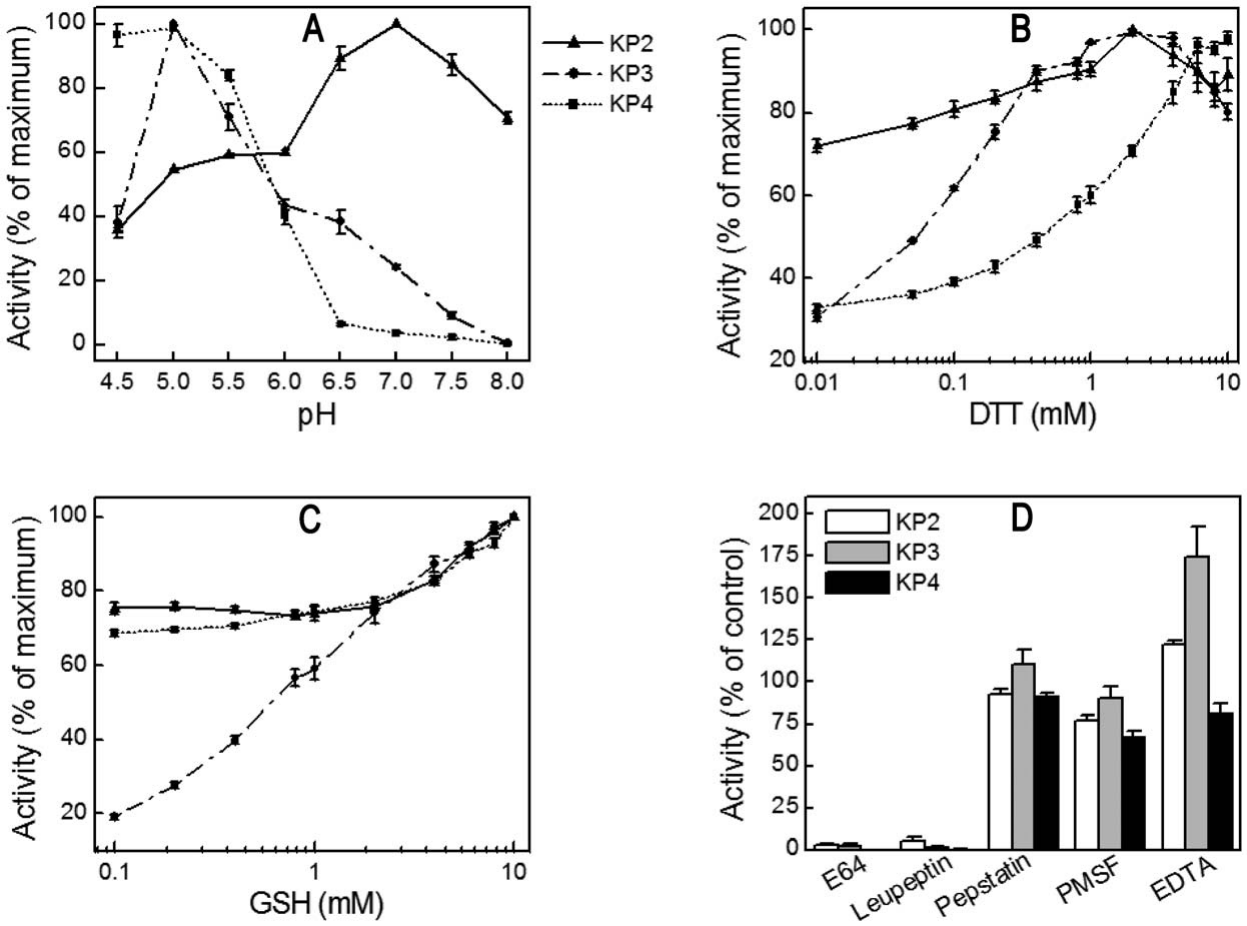
Biochemical properties of knowpains. Hydrolysis of the fluorogenic substrate Z-LR-AMC by recombinant knowpains was measured at 37°C for 30 min by monitoring the release of AMC as described in the Materials and Methods section. **A. Effect of pH.** Hydrolysis was measured in different pH buffers (100 mM sodium acetate, pH 4.5 to 6.0; 25 mM Bis-Tris, pH 6.5; and 25 mM Tris, pH 7.0 to 8.0) containing 10 mM DTT. Activities were normalized against the maximum activity to obtain % of the maximum activity and plotted against the pH. **B** and **C.** **Effect of reducing conditions.** Hydrolysis was measured in the presence of different concentrations of DTT (**B**) or GSH (**C**) in 100 mM sodium acetate pH 5.5, and activities at different concentrations were normalized with the maximum activity to obtain % of the maximum activity and plotted against the concentrations of DTT or GSH. **D. Effect of inhibitors.** Knowpains were incubated with DMSO as a control (0.75%) or indicated inhibitors (E64, leupeptin, and pepstatin, PMSF, and EDTA) in sodium acetate buffer for 5 min at room temperature. Z-LR-AMC was added and activity was measured as described in the Materials and Methods section. Activities were expressed as % of the control and plotted against corresponding inhibitors. Data shown are means ± standard error from at least three independent experiments in triplicate.

**Figure 4:**
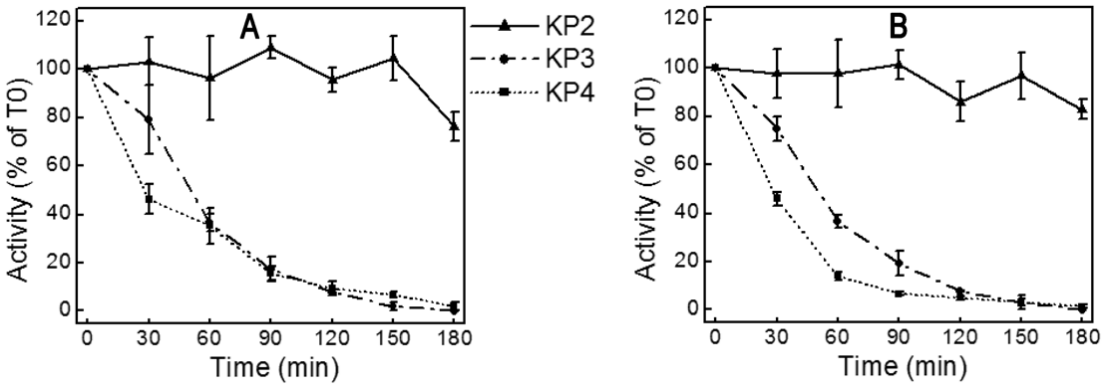
pH stability of knowpains. Knowpains were incubated at pH 5.5 (**A**) or pH 7.5 (**B**), identical aliquots were taken out in the beginning (T0) and after indicated time intervals and assessed for hydrolysis of Z-LR-AMC as described in the Materials and Methods section. Activities were expressed as % of T0 activity and plotted against time intervals (Time). Data shown are means ± standard error from three independent experiments in triplicate (except for KP3, two experiments in duplicate).

Recombinant knowpains were evaluated for hydrolysis of fluorogenic peptide substrates at different pH and reducing conditions, and for the effect of protease inhibitors. KP3 and KP4 showed maximum activity at pH 5.0; whereas KP2 showed two pH optima, with maximum activity at pH 7.0 and nearly 50% of the maximum activity at pH 5–6 (Figure 3A). The activities of all three proteases were enhanced by reducing agents and blocked by the cysteine protease inhibitors E64 and leupeptin (Figures 3B, C, and D), which are characteristic biochemical properties of papain-family proteases. Non-cysteine protease inhibitors pepstatin, PMSF, and EDTA did not inhibit knowpains; EDTA enhanced the activities of KP2 and KP3 by ∼25% and 75%, respectively. KP2 was remarkably stable at pH 5.5 and 7.5, as it retained nearly 80% of the initial activity even after 3 hour incubation at these pH conditions (Figure 4). On the other hand, under identical conditions, KP3 and KP4 lost almost 60% of the initial activity after 1 hour exposure and nearly all activity after 3 hours.

Among the three fluorogenic peptide substrates tested, KP4 hydrolyzed all three substrates, but KP2 and KP3 hydrolyzed Z-LR-AMC only (Table 1). Consistent with its maximum activity in neutral pH, KP2 hydrolyzed Z-LR-AMC more efficiently at pH 7.0 than at pH 5.5. The *k*_cat_/*K*_m_ of KP4 for Z-RR-AMC was 6.7 and 14.3 times higher than that with Z-LR-AMC and Z-FR-AMC, respectively, which makes KP4 unique among the FP2/3 subfamily proteases, as all other proteases in this sub-family have highest *k*_cat_/*K*_m_ values for Z-LR-AMC. Among knowpains, KP4 showed the highest *k*_cat_/*K*_m_ values for all three substrates, indicating that it is the most efficient enzyme, with broad substrate specificity.

**Table 1:** Kinetic parameters of knowpains for peptide substrates.

Enzyme	Substrate	*V*_max_(fentomoles.sec^−1^)	*K*_m_(µM)	*k*_cat_(Sec^−1^)	*k*_cat_/*K*_m_(Sec^−1^.M^−1^)
KP2*	Z-LR-AMC	56.50±1.19	14.26±0.27	0.0011	75.6
KP2(pH 7.0)	Z-LR-AMC	72.82±2.78	23.58±0.52	0.0029	1.23×10^2^
KP3*	Z-LR-AMC	312.36±30.74	30.56±1.23	0.0156	5.11×10^2^
KP4	Z-LR-AMC	77.39±8.07	4.20±0.25	0.0129	3.07×10^3^
KP4	Z-FR-AMC	36.91±2.49	4.20±0.19	0.0062	1.47×10^3^
KP4	Z-RR-AMC	260.93±18.80	2.13±0.17	0.0435	2.05×10^4^

Enzyme kinetics parameter value represents mean ± SE from three independent experiments performed in triplicate (two independent experiments for KP4 with Z-RR-AMC and KP3 with Z-LR-AMC performed in triplicate).

* No hydrolysis was detected with Z-FR-AMC and Z-RR-AMC.

### Knowpains Degrade Haemoglobin and Erythrocyte Cytoskeleton Proteins

**Figure 5:**
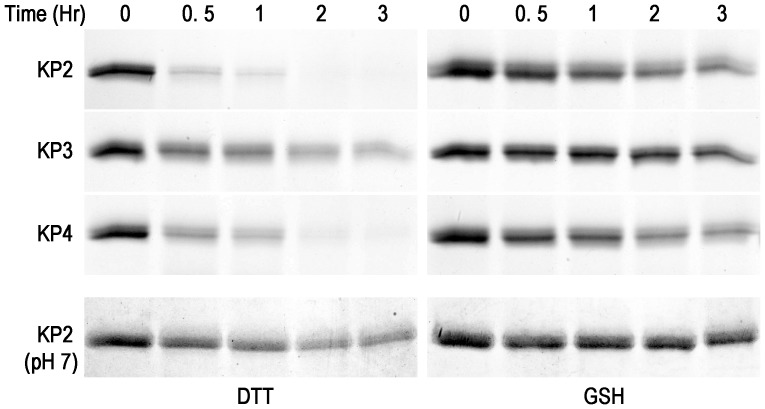
Hydrolysis of haemoglobin by knowpains. Identical concentrations (50 nM) of indicated knowpains were incubated with haemoglobin in sodium acetate buffer or Tris-Cl buffer (KP2 (pH 7)) containing DTT (5 mM) or GSH (2 mM) at 37°C. Identical aliquots were taken out at the indicated time points and reactions were stopped by adding SDS-PAGE sample buffer. Samples were resolved by 16% SDS/PAGE, and the gels were stained with coomassie blue.

The erythrocytic stage development of malaria parasites critically depends on haemoglobin degradation for supplying amino acids and maintaining osmotic stability of the infected cell. FP2/3 subfamily proteases have been shown to be major haemoglobin-degrading proteases. Hence, all three knowpains were tested for degradation of haemoglobin at the food vacuolar pH (5.5), and KP2 was also assessed for haemoglobin degradation at pH 7.0. All three proteases degraded haemoglobin at the vacuolar pH, which was more efficient in the presence of DTT than GSH (Figure 5). However, KP3 showed very weak haemoglobin hydrolysis in the presence of DTT and almost no degradation in the presence of GSH. KP4 seems to be the most efficient haemoglobinase among the three knowpains. In contrast to higher peptidolytic activity at neutral pH, KP2 degraded haemoglobin less efficiently at pH 7.0 than at pH 5.5. This result is surprising and could be due to structural changes in haemglobin at the vacuolar pH, thereby making haemoglobim more susceptible to degradation in the food vacuole.

**Figure 6:**
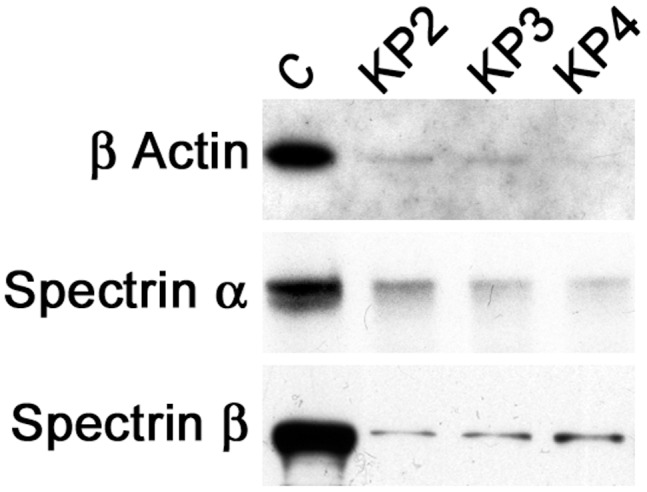
Erythrocyte cytoskeleton protein degradation by knowpains. The erythrocyte membrane preparations (30 µg) were incubated with indicated knowpains (200 nM) or without any enzyme (C) in 150 µl of 50 mM Tris-Cl, pH 7.5 for 1 hour at 37°C. The reactions were stopped by adding SDS-PAGE reducing sample buffer, identical aliquots of the samples were run in 10% SDS PAGE, transferred onto a nitrocellulose membrane, and the membrane was probed with antibodies against β actin, spectrin α, and spectrin β proteins. Significantly weaker signal in lanes corresponding to knowpains than the control lane indicates efficient degradation of all three cytoskeleton proteins by knowpains.

FP2/3 subfamily proteases appear to facilitate merozoite egress by degrading erythrocyte cytoskeleton proteins; hence, we tested knowpains for degradation of the erythrocyte cytoskeleton proteins β actin, spectrin α, and spectrin β at pH 7.5. All three knowpains efficiently degraded the three cytoskeleton proteins, and KP4 appears to be slightly more efficient than the other two enzymes, particularly with β actin and spectrin α (Figure 6).

### E64 Blocked *P. knowlesi* Erythrocytic Development

**Figure 7:**
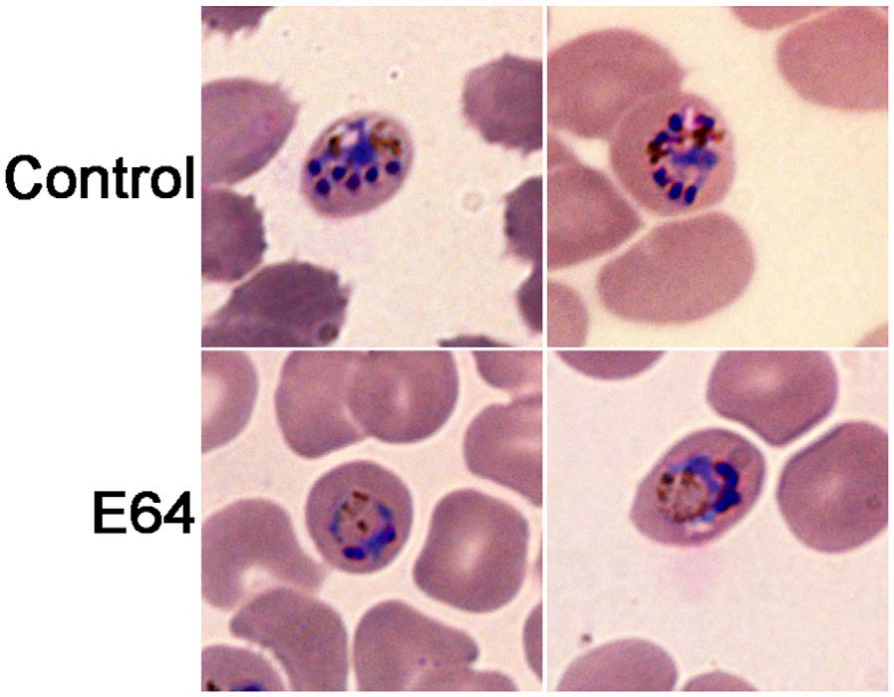
Effect of E64 on *P. knowlesi* development. Ring stage parasites were cultured without (control) or with E64 (12.5 µM) for 20 hours, thin smears were prepared on glass slides, stained with Giemsa, the morphologies of parasites were examined under a 100× objective lens using a Zeiss AxioImager Z1 microscope. Note that the inhibitor-treated parasites have enlarged food vacuoles containing the food vacuole-resident pigment haemozoin, which are not so obvious in controls.

Cysteine protease inhibitors like E64 block the development of *P. falciparum*, with enlargement of the food vacuole. A large body of evidence, including knockout of FP2, indicates that the food vacuole phenotype is a result of accumulation of undegraded haemoglobin in the food vacuole upon inhibition or loss of falcipains. To determine if *P. knowlesi* is susceptible to cysteine protease inhibitors, blood stage parasites were cultured without or with E64 and maturation, growth rates, and morphology were determined. E64 blocked *P. knowlesi* maturation (IC_50_ = 5.4 µM), and the treated parasites developed swollen food vacuoles (Figure 7), indicating a block in haemoglobin degradation. E64 completely blocked parasite multiplication at concentration ≥1.56 µM, a result similar to that seen in in vitro studies of *P. falciparum* [7].

### Knowpains are Expressed in Erythrocytic Stage *P. knowlesi* Parasites

**Figure 8:**
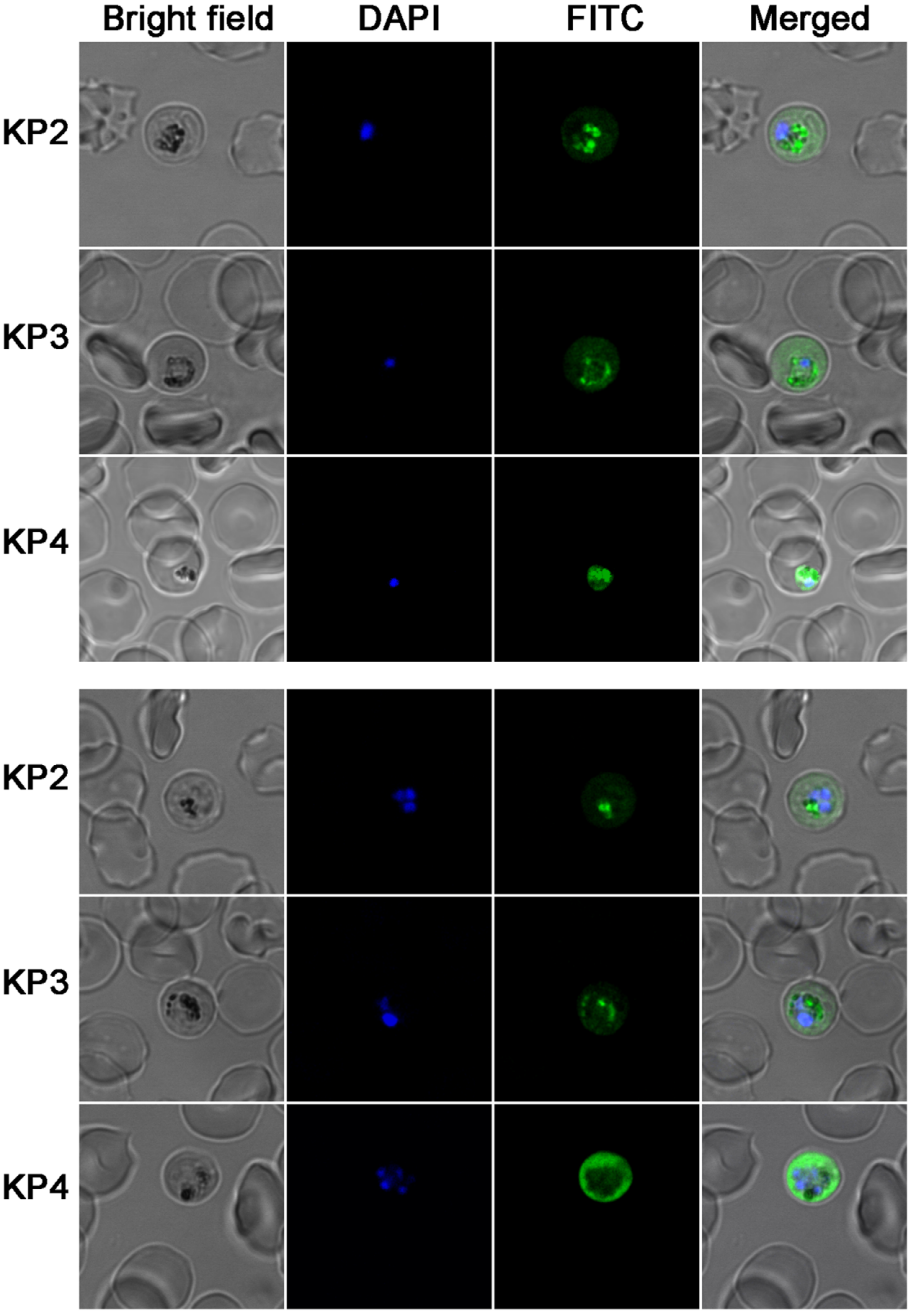
Expression of knowpains in *P. knowlesi* . Late trophozoite and schizont stages of *P. knowlesi* were immobilized on poly-Lysine coated slides, fixed, permeabilized RBCs, probed with antibodies against indicated knowpains and then with FITC conjugated-anti-rat IgG, and stained with the nuclear stain DAPI. Shown are the bright field, staining of the nucleus (DAPI), knowpain signal (FITC), and overlap of the three images (merged). Figures indicate expression of all three knowpains in trophozoite (top panel) and schizont (bottom panel) stage parasites. Note that KP2 and KP3 predominantly colocalized with the black food vacuole-resident malaria pigment haemozoin, indicating their presence in the food vacuole. KP4 signal is markedly stronger towards the parasite periphery and is also discernible in the areas containing haemozoin, indicating the presence of KP4 both in the food vacuole and the cytoplasm.

As recombinant knowpains showed biochemical properties required for a protease to degrade haemoglobin in the food vacuole, their expression and cellular localization were determined in trophozoite and schizont stages of *P. knowlesi* by IFA using enzyme-specific antisera. All three knowpains were expressed in erythrocytic stage parasites, and nearly identical localization pattern was observed for each protein in trophozoite and schizont stages (Figure 8). KP2 and KP3 were predominantly colocalized with the food vacuole-resident pigment haemozoin, indicating their presence in the food vacuole. The KP4 signal was throughout the parasite, including the food vacuole, but markedly stronger towards the parasite periphery.

## Discussion

*P. knowlesi*, which was considered mainly a monkey malaria parasite until recently, has been established as the fifth human malaria parasite, with potential to cause severe and fatal malaria in humans [2], [3]. The current study reports expression, biochemical characterization, and cellular localization of three FP2/3 subfamily papain-like cysteine proteases of *P. knowlesi*, KP2, KP3, and KP4. Characterization of knowpains represents an important advance to our current understanding of these leading drug targets of malaria parasites. Sequence analysis categorized the three knowpains as FP2/3 subfamily papain-like cysteine proteases as, like FP2 and FP3, they contain longer prodomains than most papain-like proteases, an N-terminal refolding domain and a C-terminal insert in mature domains. Knowpains are most similar to vivapains, and could be separated into homologs of individual vivapains.

Knowpains share several biochemical similarities with other characterized FP2/3 subfamily proteases, including maximum (KP3 and KP4) or modest (KP2) activity near the food vacuolar pH, enhanced activity under reducing conditions, inhibition by cysteine protease inhibitors, and degradation of haemoglobin [10]. This indicates that knowpains are competent haemoglobinases. However, knowpains showed several obvious differences in biochemical properties, both among the three proteases and when compared to falcipains or vivapains. Unlike FP2 and FP3, but similar to VX2 and VX3, KP2 and KP3 did not hydrolyze Z-FR-AMC and Z-RR-AMC [13], [27], [28]. KP4, like VX4 and FP2, hydrolyzed all three peptide substrates tested at pH 5.5 but preferred Arg>Leu>Phe residues at the P2 position in substrates whereas both FP2 and VX4 favor Leu>Phe>Arg [14], [27]. Importantly, the P2 leucine selectivity has also been demonstrated for inhibitors in the cases of FP2, FP3, VX2, and VX3 [13], which is consistent with over 20 times greater inhibition of parasite growth by compounds with P2 leucine compared to those with P2 phenylalanine [54]. Furthermore, KP2, unlike KP3 and KP4, showed more than 50% of maximum activity in a wide pH range (5.0–8.0), and it was remarkably stable at pH 5.5 and 7.5. The pH and stability profiles of KP2 are similar to those of VX2, but differ from the rest of the studied FP2/3 subfamily proteases. Thus, the absolute preference of KP2 and KP3 and modest preference of KP4 for P2 leucine suggest that available falcipain inhibitors might be effective against knowpains, and argue for designing effective common inhibitors of the FP2/3 subfamily proteases as treatments for malaria.

Subcellular localization experiments indicated the presence of KP2 and KP3 predominantly in the food vacuole, as they colocalized with the food vacuole-resident pigment haemozoin. KP4, on the other hand, was predominantly present in the cytoplasm towards the parasite periphery, but also colocalized with haemozoin, indicating its presence in the food vacuole as well. The presence of all three knowpains in the food vacuole together with their ability to degrade haemoglobin under acidic conditions supports their function as haemoglobinases. In addition to haemoglobin degradation, inhibition of merozoite egress from mature schizont-infected erythrocytes by E64 has suggested degradation of erythrocyte cytoskeleton proteins by falcipains [35], [36]. KP4 may also have a role in cleavage of the erythrocyte cytoskeleton proteins to facilitate merozoite egress; as it is predominantly present towards the parasite periphery, has broad substrate specificity, and it efficiently degraded erythrocyte cytoskeleton proteins in vitro. KP2, owing to its activity and stability in a broad pH range and ability to degrade erythrocyte cytoskeleton proteins and discernible presence in the parasite cytoplasm, seems to be another candidate for degradation of erythrocyte cytoskeleton proteins. However, predominant food vacuolar localization and extremely low activity against only P2 leucine-containing substrates suggest that its main function is to degrade haemoglobin, which is a leucine rich protein (α unit: 12.7% leucine, β unit: 12.2% leucine). Similarly, KP3 appears to be chiefly suited for degradation of haemoglobin, as it is primarily present in the food vacuole, maximally active in acidic pH, and degrades P2 leucine-containing substrates only. KP4, based on its localization both in the food vacuole and the parasite cytoplasm, broad substrate specificity, and most efficient degradation of haemoglobin and erythrocyte cytoskeleton proteins among knowpains, likely has roles both in haemoglobin hydrolysis and merozoite egress. Subcellular localization of knowpains adds to their functional relatedness with FP2 and FP3, as FP2 is exclusively present in the food vacuole and FP3 is predominantly present in the food vacuole but also present in the parasite cytoplasm as punctate structures [55].

A comparative analysis of sequences and biochemical properties of knowpains and vivapains revealed a 1 to 1 relationship between different homologs (Table S1). For example, VX4 and KP4 share at least 20% more sequence identity with each other than with any other protease of the family; both are optimally active in acid pH (5.0–6.0), degrade all three peptide substrates tested, and are present in the parasite food vacuole and cytoplasm. Similarly, KP2 and KP3 are most similar to VX2 and VX3, respectively. This information suggests that VX2 and VX3 are food vacuole haemoglobinases, though localization studies have not been done for these enzymes. Furthermore, striking biochemical and cellular similarities between knowpains and vivapains (at least between KP4 and VX4) supports the use of in vitro culture of *P. knowlesi* for testing vivapain-specific inhibitors in the absence of an efficient in vitro culture system for *P. vivax*.

Several studies demonstrate that inhibitors of papain-like cysteine proteases, like E64, block the erythrocytic stage development of *P. falciparum* in culture and cure mice infected with the mouse malaria parasite *P. vinckei* [7], [9]. Considering similarities between different parasite species, it is logical to assume that cysteine protease inhibitors will block the development of all malaria parasites and thus that the papain-like cysteine proteases of human malaria parasites are promising targets for development of new antimalarials. To validate knowpains as potential drug targets, *P. knowlesi* was cultured in the presence of E64. As has been seen with *P. falciparum*, E64 inhibited the erythrocytic stage development of *P. knowlesi*, which was accompanied with enlargement of the food vacuole. The enlargement of the food vacuole is an indication of a block in haemoglobin degradation due to inhibition of knowpains and supports the drug target potential of knowpains. Thus, ongoing falcipain-based drug discovery programs should also consider knowpains. Functional expression and characterization of knowpains enables simultaneous screening of available falcipain-inhibitor libraries against knowpains, allowing efficient screening for broadly effective compounds active against multiple species of human malaria parasites.

## Supporting Information

Figure S1 **Processing of KP3.** The coomassie-stained gel shows progress of KP3 processing. Refolded KP3 was processed at 37°C for 4 hours as described in Materials and Methods section; identical aliquots were collected at indicated time intervals, and the remaining processed sample after 4 hours was used to purify KP3 using Q-Sepharose. The aliquots, flow through (FT), and the eluate (E) of the Q-Sepharose purification were run on 12% SDS-PAGE. The lower band near 14 kDa is the thioredoxin/His-tag, which is absent in the eluate sample.
(TIF)

Figure S2 **Cross reactivity of knowpain antisera.** The indicated recombinant mature knowpains were used in western blots (anti-KP2 and anti-KP4) and dot (anti-KP3) to assess cross reactivity of antisera. For western, proteins (1 µg) were resolved on 12% SDS-PAGE and transferred onto the nitrocellulose membrane (Millipore). For dot blot, 0.5 µg of each protein was spotted on the nitrocellulose membrane and allowed to dry at room temperature. The membranes were blocked (PBS with 0.1% Tween 20 and 3% BSA) for 1 hour at room temperature, incubated with knowpain antisera (anti-KP2 at 1/1000, anti-KP3 at 1/1000, and anti-KP4 at 1/500 dilution), washed with blocking buffer, and incubated with HRP-conjugated goat anti-rabbit IgG (Invitrogen; at 1/10000 dilution in blocking buffer) for 1 hour at room temperature. The membranes were washed with blocking buffer and the signal for western blot was developed with the SuperSignal West Pico Chemiluminescent kit (Pierce) on X-ray film (Pierce); the dot blot signal was developed using the Novex® HRP Chromogenic Substrate (Invitrogen). Each antiserum strongly reacted with the corresponding knowpain but not with other two knowpains, indicating that the sera are specific.
(TIF)

Table S1 **Comparison of the FP2/3 subfamily proteases of human malaria parasites.**
(DOCX)

## References

[1] WHO (2011) The World malaria report 2011. The World Health Organization.

[2] Singh B, Kim Sung L, Matusop A, Radhakrishnan A, Shamsul SS, et al (2004) A large focus of naturally acquired *Plasmodium knowlesi* infections in human beings. Lancet 363: 1017–1024. 15051281 10.1016/S0140-6736(04)15836-4

[3] Daneshvar C, Davis TM, Cox-Singh J, Rafa’ee MZ, Zakaria SK, et al (2009) Clinical and laboratory features of human *Plasmodium knowlesi* infection. Clin Infect Dis 49: 852–860. 19635025 10.1086/605439 PMC2843824

[4] Cox-Singh J, Davis TM, Lee KS, Shamsul SS, Matusop A, et al (2008) *Plasmodium knowlesi* malaria in humans is widely distributed and potentially life threatening. Clin Infect Dis 46: 165–171. 18171245 10.1086/524888 PMC2533694

[5] Gelb MH (2007) Drug discovery for malaria: a very challenging and timely endeavor. Curr Opin Chem Biol 11: 440–445. 17761335 10.1016/j.cbpa.2007.05.038 PMC1993815

[6] Muller IB, Hyde JE (2010) Antimalarial drugs: modes of action and mechanisms of parasite resistance. Future Microbiol 5: 1857–1873. 21155666 10.2217/fmb.10.136

[7] Rosenthal PJ, McKerrow JH, Aikawa M, Nagasawa H, Leech JH (1988) A malarial cysteine proteinase is necessary for hemoglobin degradation by *Plasmodium falciparum* . J Clin Invest 82: 1560–1566. 3053784 10.1172/JCI113766 PMC442723

[8] Rosenthal PJ, Wollish WS, Palmer JT, Rasnick D (1991) Antimalarial effects of peptide inhibitors of a *Plasmodium falciparum* cysteine proteinase. J Clin Invest 88: 1467–1472. 1939639 10.1172/JCI115456 PMC295650

[9] Rosenthal PJ, Lee GK, Smith RE (1993) Inhibition of a *Plasmodium vinckei* cysteine proteinase cures murine malaria. J Clin Invest 91: 1052–1056. 8450035 10.1172/JCI116262 PMC288059

[10] Rosenthal PJ (2011) Falcipains and other cysteine proteases of malaria parasites. Adv Exp Med Biol 712: 30–48. 21660657 10.1007/978-1-4419-8414-2_3

[11] Marco M, Coteron JM (2012) Falcipain inhibition as a promising antimalarial target. Curr Top Med Chem 12: 408–444. 22242849 10.2174/156802612799362913

[12] Ettari R, Bova F, Zappala M, Grasso S, Micale N (2010) Falcipain-2 inhibitors. Med Res Rev 30: 136–167. 19526594 10.1002/med.20163

[13] Na BK, Shenai BR, Sijwali PS, Choe Y, Pandey KC, et al (2004) Identification and biochemical characterization of vivapains, cysteine proteases of the malaria parasite *Plasmodium vivax* . Biochem J 378: 529–538. 14629194 10.1042/BJ20031487 PMC1223978

[14] Na BK, Bae YA, Zo YG, Choe Y, Kim SH, et al (2010) Biochemical properties of a novel cysteine protease of *Plasmodium vivax*, vivapain-4. PLoS Negl Trop Dis 4: e849. 20967286 10.1371/journal.pntd.0000849 PMC2953480

[15] Brooks SR, Williamson KC (2000) Proteolysis of *Plasmodium falciparum* surface antigen, Pfs230, during gametogenesis. Mol Biochem Parasitol 106: 77–82. 10743612 10.1016/s0166-6851(99)00201-7

[16] Eksi S, Czesny B, van Gemert GJ, Sauerwein RW, Eling W, et al (2007) Inhibition of *Plasmodium falciparum* oocyst production by membrane-permeant cysteine protease inhibitor E64d. Antimicrob Agents Chemother 51: 1064–1070. 17178799 10.1128/AAC.01012-06 PMC1803139

[17] Rupp I, Bosse R, Schirmeister T, Pradel G (2008) Effect of protease inhibitors on exflagellation in *Plasmodium falciparum* . Mol Biochem Parasitol 158: 208–212. 18243365 10.1016/j.molbiopara.2007.12.009

[18] Coppi A, Pinzon-Ortiz C, Hutter C, Sinnis P (2005) The Plasmodium circumsporozoite protein is proteolytically processed during cell invasion. J Exp Med 201: 27–33. 15630135 10.1084/jem.20040989 PMC1995445

[19] Goldberg DE (2005) Hemoglobin degradation. Curr Top Microbiol Immunol 295: 275–291. 16265895 10.1007/3-540-29088-5_11

[20] Lew VL, Tiffert T, Ginsburg H (2003) Excess hemoglobin digestion and the osmotic stability of *Plasmodium falciparum*-infected red blood cells. Blood 101: 4189–4194. 12531811 10.1182/blood-2002-08-2654

[21] Krugliak M, Zhang J, Ginsburg H (2002) Intraerythrocytic *Plasmodium falciparum* utilizes only a fraction of the amino acids derived from the digestion of host cell cytosol for the biosynthesis of its proteins. Mol Biochem Parasitol 119: 249–256. 11814576 10.1016/s0166-6851(01)00427-3

[22] Sijwali PS, Koo J, Singh N, Rosenthal PJ (2006) Gene disruptions demonstrate independent roles for the four falcipain cysteine proteases of *Plasmodium falciparum* . Mol Biochem Parasitol 150: 96–106. 16890302 10.1016/j.molbiopara.2006.06.013

[23] Eggleson KK, Duffin KL, Goldberg DE (1999) Identification and characterization of falcilysin, a metallopeptidase involved in hemoglobin catabolism within the malaria parasite *Plasmodium falciparum* . J Biol Chem 274: 32411–32417. 10542284 10.1074/jbc.274.45.32411

[24] Banerjee R, Liu J, Beatty W, Pelosof L, Klemba M, et al (2002) Four plasmepsins are active in the *Plasmodium falciparum* food vacuole, including a protease with an active-site histidine. Proc Natl Acad Sci U S A 99: 990–995. 11782538 10.1073/pnas.022630099 PMC117418

[25] Klemba M, Gluzman I, Goldberg DE (2004) A *Plasmodium falciparum* dipeptidyl aminopeptidase I participates in vacuolar hemoglobin degradation. J Biol Chem 279: 43000–43007. 15304495 10.1074/jbc.M408123200

[26] Sijwali PS, Rosenthal PJ (2004) Gene disruption confirms a critical role for the cysteine protease falcipain-2 in hemoglobin hydrolysis by *Plasmodium falciparum* . Proc Natl Acad Sci U S A 101: 4384–4389. 15070727 10.1073/pnas.0307720101 PMC384756

[27] Shenai BR, Sijwali PS, Singh A, Rosenthal PJ (2000) Characterization of native and recombinant falcipain-2, a principal trophozoite cysteine protease and essential hemoglobinase of *Plasmodium falciparum* . J Biol Chem 275: 29000–29010. 10887194 10.1074/jbc.M004459200

[28] Sijwali PS, Shenai BR, Gut J, Singh A, Rosenthal PJ (2001) Expression and characterization of the *Plasmodium falciparum* haemoglobinase falcipain-3. Biochem J 360: 481–489. 11716777 10.1042/0264-6021:3600481 PMC1222249

[29] Rosenthal PJ, Nelson RG (1992) Isolation and characterization of a cysteine proteinase gene of *Plasmodium falciparum* . Mol Biochem Parasitol 51: 143–152. 1565129 10.1016/0166-6851(92)90209-3

[30] Singh N, Sijwali PS, Pandey KC, Rosenthal PJ (2006) *Plasmodium falciparum*: biochemical characterization of the cysteine protease falcipain-2′. Exp Parasitol 112: 187–192. 16337629 10.1016/j.exppara.2005.10.007

[31] Salas F, Fichmann J, Lee GK, Scott MD, Rosenthal PJ (1995) Functional expression of falcipain, a *Plasmodium falciparum* cysteine proteinase, supports its role as a malarial hemoglobinase. Infect Immun 63: 2120–2125. 7768590 10.1128/iai.63.6.2120-2125.1995 PMC173275

[32] Jeong JJ, Kumar A, Hanada T, Seo PS, Li X, et al (2006) Cloning and characterization of *Plasmodium falciparum* cysteine protease, falcipain-2B. Blood Cells Mol Dis 36: 429–435. 16595182 10.1016/j.bcmd.2006.02.003

[33] Dluzewski AR, Rangachari K, Wilson RJ, Gratzer WB (1986) *Plasmodium falciparum*: protease inhibitors and inhibition of erythrocyte invasion. Exp Parasitol 62: 416–422. 3536568 10.1016/0014-4894(86)90050-0

[34] Hadley T, Aikawa M, Miller LH (1983) *Plasmodium knowlesi*: studies on invasion of rhesus erythrocytes by merozoites in the presence of protease inhibitors. Exp Parasitol 55: 306–311. 6852169 10.1016/0014-4894(83)90027-9

[35] Dhawan S, Dua M, Chishti AH, Hanspal M (2003) Ankyrin peptide blocks falcipain-2-mediated malaria parasite release from red blood cells. J Biol Chem 278: 30180–30186. 12775709 10.1074/jbc.M305132200

[36] Dua M, Raphael P, Sijwali PS, Rosenthal PJ, Hanspal M (2001) Recombinant falcipain-2 cleaves erythrocyte membrane ankyrin and protein 4.1. Mol Biochem Parasitol 116: 95–99. 11463472 10.1016/s0166-6851(01)00306-1

[37] Sijwali PS, Kato K, Seydel KB, Gut J, Lehman J, et al (2004) *Plasmodium falciparum* cysteine protease falcipain-1 is not essential in erythrocytic stage malaria parasites. Proc Natl Acad Sci U S A 101: 8721–8726. 15166288 10.1073/pnas.0402738101 PMC423262

[38] Greenbaum DC, Baruch A, Grainger M, Bozdech Z, Medzihradszky KF, et al (2002) A role for the protease falcipain 1 in host cell invasion by the human malaria parasite. Science 298: 2002–2006. 12471262 10.1126/science.1077426

[39] Eksi S, Czesny B, Greenbaum DC, Bogyo M, Williamson KC (2004) Targeted disruption of *Plasmodium falciparum* cysteine protease, falcipain 1, reduces oocyst production, not erythrocytic stage growth. Mol Microbiol 53: 243–250. 15225318 10.1111/j.1365-2958.2004.04108.x

[40] Wang SX, Pandey KC, Somoza JR, Sijwali PS, Kortemme T, et al (2006) Structural basis for unique mechanisms of folding and hemoglobin binding by a malarial protease. Proc Natl Acad Sci U S A 103: 11503–11508. 16864794 10.1073/pnas.0600489103 PMC1544199

[41] Kerr ID, Lee JH, Pandey KC, Harrison A, Sajid M, et al (2009) Structures of falcipain-2 and falcipain-3 bound to small molecule inhibitors: implications for substrate specificity. J Med Chem 52: 852–857. 19128015 10.1021/jm8013663 PMC2651692

[42] Hogg T, Nagarajan K, Herzberg S, Chen L, Shen X, et al (2006) Structural and functional characterization of Falcipain-2, a hemoglobinase from the malarial parasite *Plasmodium falciparum* . J Biol Chem 281: 25425–25437. 16777845 10.1074/jbc.M603776200

[43] Choe Y, Leonetti F, Greenbaum DC, Lecaille F, Bogyo M, et al (2006) Substrate profiling of cysteine proteases using a combinatorial peptide library identifies functionally unique specificities. J Biol Chem 281: 12824–12832. 16520377 10.1074/jbc.M513331200

[44] Subramanian S, Hardt M, Choe Y, Niles RK, Johansen EB, et al (2009) Hemoglobin cleavage site-specificity of the *Plasmodium falciparum* cysteine proteases falcipain-2 and falcipain-3. PLoS One 4: e5156. 19357776 10.1371/journal.pone.0005156 PMC2663817

[45] Ramjee MK, Flinn NS, Pemberton TP, Quibell M, Wang Y, et al (2006) Substrate mapping and inhibitor profiling of falcipain-2, falcipain-3 and berghepain-2: implications for peptidase anti-malarial drug discovery. Biochem J 399: 47–57. 16776649 10.1042/BJ20060422 PMC1570174

[46] Singh A, Shenai BR, Choe Y, Gut J, Sijwali PS, et al (2002) Critical role of amino acid 23 in mediating activity and specificity of vinckepain-2, a papain-family cysteine protease of rodent malaria parasites. Biochem J 368: 273–281. 12169096 10.1042/BJ20020753 PMC1222974

[47] Singh A, Walker KJ, Sijwali PS, Lau AL, Rosenthal PJ (2007) A chimeric cysteine protease of *Plasmodium berghei* engineered to resemble the *Plasmodium falciparum* protease falcipain-2. Protein Eng Des Sel 20: 171–177. 17430972 10.1093/protein/gzm009

[48] Escalante AA, Ayala FJ (1995) Evolutionary origin of Plasmodium and other Apicomplexa based on rRNA genes. Proc Natl Acad Sci U S A 92: 5793–5797. 7597031 10.1073/pnas.92.13.5793 PMC41587

[49] Kocken CH, Ozwara H, van der Wel A, Beetsma AL, Mwenda JM, et al (2002) *Plasmodium knowlesi* provides a rapid in vitro and in vivo transfection system that enables double-crossover gene knockout studies. Infect Immun 70: 655–660. 11796595 10.1128/IAI.70.2.655-660.2002 PMC127703

[50] Mayxay M, Pukrittayakamee S, Newton PN, White NJ (2004) Mixed-species malaria infections in humans. Trends Parasitol 20: 233–240. 15105024 10.1016/j.pt.2004.03.006

[51] Karrer KM, Peiffer SL, DiTomas ME (1993) Two distinct gene subfamilies within the family of cysteine protease genes. Proc Natl Acad Sci U S A 90: 3063–3067. 8464925 10.1073/pnas.90.7.3063 PMC46237

[52] Coulombe R, Grochulski P, Sivaraman J, Menard R, Mort JS, et al (1996) Structure of human procathepsin L reveals the molecular basis of inhibition by the prosegment. EMBO J 15: 5492–5503. 8896443 PMC452294

[53] Pandey KC, Barkan DT, Sali A, Rosenthal PJ (2009) Regulatory elements within the prodomain of Falcipain-2, a cysteine protease of the malaria parasite *Plasmodium falciparum* . PLoS One 4: e5694. 19479029 10.1371/journal.pone.0005694 PMC2682653

[54] Rosenthal PJ, Olson JE, Lee GK, Palmer JT, Klaus JL, et al (1996) Antimalarial effects of vinyl sulfone cysteine proteinase inhibitors. Antimicrob Agents Chemother 40: 1600–1603. 8807047 10.1128/aac.40.7.1600 PMC163380

[55] Subramanian S, Sijwali PS, Rosenthal PJ (2007) Falcipain cysteine proteases require bipartite motifs for trafficking to the *Plasmodium falciparum* food vacuole. J Biol Chem 282: 24961–24969. 17565983 10.1074/jbc.M703316200

