# Supplementary figures and images for: Expression, Characterization, and Cellular Localization of Knowpains, Papain-Like Cysteine Proteases of the Plasmodium knowlesi Malaria Parasite
Source: PLoS One. 2012 Dec 12;7(12):e51619. doi: 10.1371/journal.pone.0051619 (PMC3520923; doi:10.1371/journal.pone.0051619)

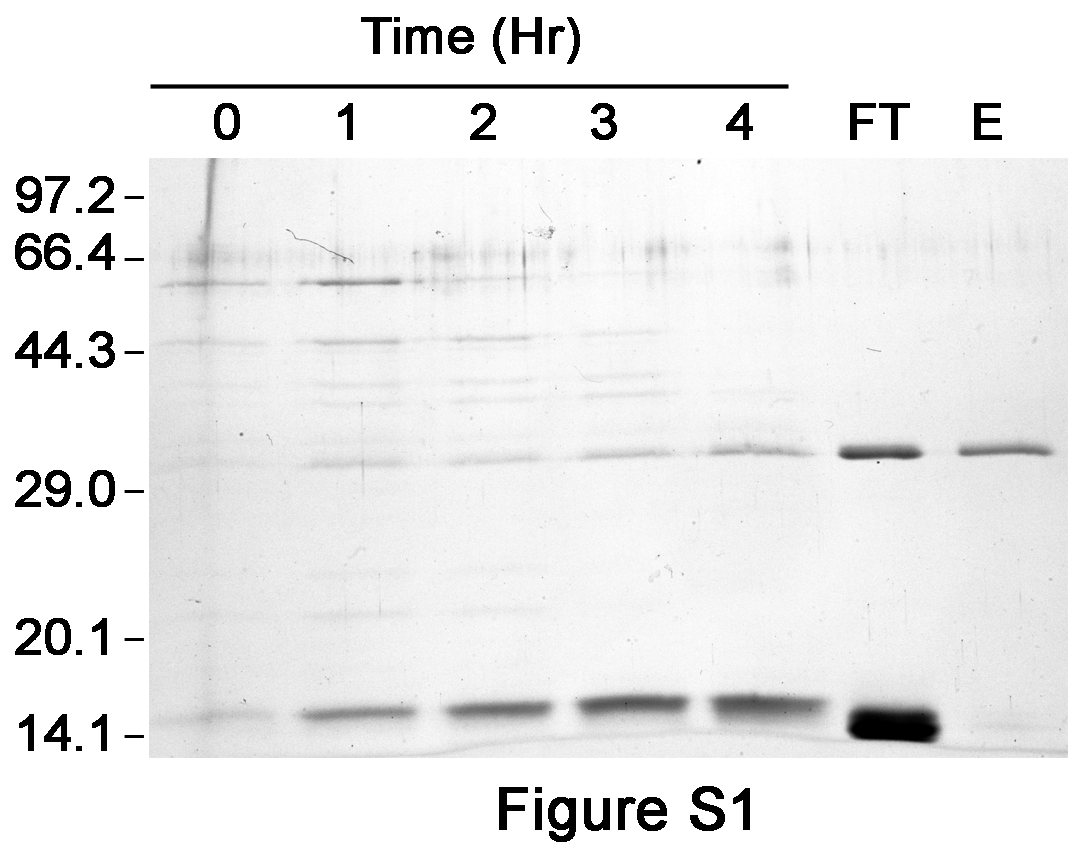

Supplement: Figure S1 — Processing of KP3. The coomassie-stained gel shows progress of KP3 processing. Refolded KP3 was processed at 37°C for 4 hours as described in Materials and Methods section; identical aliquots were collected at indicated time intervals, and the remaining processed sample after 4 hours was used to purify KP3 using Q-Sepharose. The aliquots, flow through (FT), and the eluate (E) of the Q-Sepharose purification were run on 12% SDS-PAGE. The lower band near 14 kDa is the thioredoxin/His-tag, which is absent in the eluate sample. (TIF) [file pone.0051619.s001.tif]

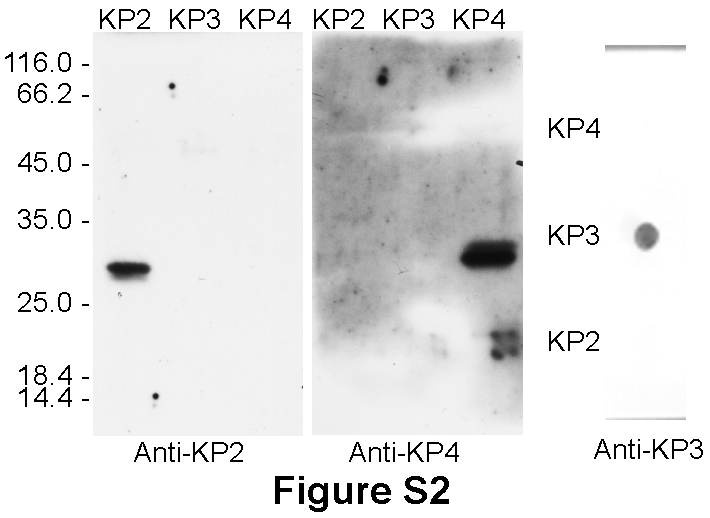

Supplement: Figure S2 — Cross reactivity of knowpain antisera. The indicated recombinant mature knowpains were used in western blots (anti-KP2 and anti-KP4) and dot (anti-KP3) to assess cross reactivity of antisera. For western, proteins (1 µg) were resolved on 12% SDS-PAGE and transferred onto the nitrocellulose membrane (Millipore). For dot blot, 0.5 µg of each protein was spotted on the nitrocellulose membrane and allowed to dry at room temperature. The membranes were blocked (PBS with 0.1% Tween 20 and 3% BSA) for 1 hour at room temperature, incubated with knowpain antisera (anti-KP2 at 1/1000, anti-KP3 at 1/1000, and anti-KP4 at 1/500 dilution), washed with blocking buffer, and incubated with HRP-conjugated goat anti-rabbit IgG (Invitrogen; at 1/10000 dilution in blocking buffer) for 1 hour at room temperature. The membranes were washed with blocking buffer and the signal for western blot was developed with the SuperSignal West Pico Chemiluminescent kit (Pierce) on X-ray film (Pierce); the dot blot signal was developed using the Novex® HRP Chromogenic Substrate (Invitrogen). Each antiserum strongly reacted with the corresponding knowpain but not with other two knowpains, indicating that the sera are specific. (TIF) [file pone.0051619.s002.tif]
