# Supplementary material for: Expression, Characterization, and Cellular Localization of Knowpains, Papain-Like Cysteine Proteases of the Plasmodium knowlesi Malaria Parasite
Source: PLoS One. 2012 Dec 12;7(12):e51619. doi: 10.1371/journal.pone.0051619 (PMC3520923; doi:10.1371/journal.pone.0051619)
Supplement: Table S1 — Comparison of the FP2/3 subfamily proteases of human malaria parasites. (DOCX) [file pone.0051619.s003.docx]

**Table S1.** Comparison of the FP2/3 subfamily proteases of human malaria parasites.

| **Proteases** | | **^1^FP2** | **^1^FP2ʹ** | **^1^FP3** | **^1^VX2** | **^1^VX3** | **^1^VX4** | **KP2** | **KP3** | **KP4** |
| --- | --- | --- | --- | --- | --- | --- | --- | --- | --- | --- |
| % Identity with knowpains | KP2 | 45 | 45 | 52 | 72 | 53 | 53 | 100 | 57 | 66 |
|  | KP3 | 45 | 46 | 50 | 53 | 78 | 48 | 57 | 100 | 50 |
|  | KP4 | 44.0 | 45 | 52 | 58 | 50 | 79 | 66 | 50 | 100 |
| Optimum pH (>90% of maximum activity) | | 5.0-6.0 | 5.5-6.5 | 5.5-6.5 | 5.5-7.0 | 5.5-6.0 | 5.0-6.0 | 6.5-7.5 | 5.0 | 4.0-5.0 |
| DTT/GSH dependent activity | | Yes | Yes | Yes | Yes | Yes | Yes | Yes | Yes | Yes |
| Hb hydrolysis at pH 5.5 | | Yes | Yes | Yes | Yes | Yes | Yes | Yes | Yes | Yes |
| Preference for P2 residue in substrates (pH 5.5) | | Leu>Phe>Arg | Leu>Phe ^a^ | Leu>Phe ^a^ | Leu^b^ | Leu^b^ | Leu>Phe>Arg | Leu^b^ | Leu^b^ | Arg>Leu>Phe |
| Sub-cellular localization | | FV | ND | FV and Cyt | ND | ND | FV and Cyt | FV | FV | FV and Cyt |
| Degradation of erythrocyte cytoskeleton proteins (pH 7.5) | | Ankyrin and  protein 4.1 | ND | ND | Actin and Band 3 | Actin and Band 3 | Actin and Band 3 | β Actin, spectrin α, and spectrin β | β Actin, spectrin α, and spectrin β | β Actin, spectrin α, and spectrin β |
| Refolding domain | | ^c^Yes | Yes^d^ | Yes^d^ | Yes^d^ | Yes^d^ | Yes^d^ | Yes^d^ | Yes^d^ | Yes^d^ |
| Hb-binding domain | | ^c^Yes | Yes^d^ | Yes^d^ | Yes^d^ | Yes^d^ | Yes^d^ | Yes^d^ | Yes^d^ | Yes^d^ |

^1^Data are from references [13, 14, 27, 28, 30]; ND, not determined; FV, food vacuole;Cyt, cytoplasm; ^a^data not available for Z-RR-AMC; ^b^did not degrade Z-FR-AMC and Z-RR-AMC; ^c^experimentally validated; ^d^putative.
